# Thyroid Cancer Stem-Like Cells: From Microenvironmental Niches to Therapeutic Strategies

**DOI:** 10.3390/jcm10071455

**Published:** 2021-04-01

**Authors:** Elisa Stellaria Grassi, Viola Ghiandai, Luca Persani

**Affiliations:** 1 Department of Medical Biotechnology and Translational Medicine, University of Milan, 20129 Milan, Italy; viola.ghiandai@unimi.it (V.G.); luca.persani@unimi.it (L.P.); 2 Laboratory of Endocrine and Metabolic Research, Istituto Auxologico Italiano IRCCS, 20149 Milan, Italy

**Keywords:** thyroid cancer, cancer stem cells, tumor microenvironment, CSCs niche, targeted therapy

## Abstract

Thyroid cancer (TC) is the most common endocrine malignancy. Recent progress in thyroid cancer biology revealed a certain degree of intratumoral heterogeneity, highlighting the coexistence of cellular subpopulations with distinct proliferative capacities and differentiation abilities. Among those subpopulations, cancer stem-like cells (CSCs) are hypothesized to drive TC heterogeneity, contributing to its metastatic potential and therapy resistance. CSCs principally exist in tumor areas with specific microenvironmental conditions, the so-called stem cell niches. In particular, in thyroid cancer, CSCs’ survival is enhanced in the hypoxic niche, the immune niche, and some areas with specific extracellular matrix composition. In this review, we summarize the current knowledge about thyroid CSCs, the tumoral niches that allow their survival, and the implications for TC therapy.

## 1. Introduction

Thyroid cancer (TC) is the most common endocrine malignancy [1]. The thyroid gland is a complex endocrine organ that is potentially affected by a variety of cancers that differ in molecular profile, morphology, tumorigenicity, and invasiveness [1]. Follicular cells can give rise to three different subtypes of thyroid carcinoma: papillary thyroid carcinoma (PTC), follicular thyroid carcinoma (FTC), poorly differentiated thyroid carcinoma (PDTC) and anaplastic thyroid carcinoma (ATC) [2]. PTCs and FTCs account for 80–85% and 10–15% of all TCs, respectively, and usually have a good prognosis. In contrast, ATCs are rare but are characterized by an aggressive phenotype and a poor prognosis [2]. Although ATCs represent only 1% of all thyroid cancers, they account for >50% of the mortality, as they often acquire therapy resistance [3,4]. TCs have a complex genetic background, with the acquisition of hyperactivating mutations in the BRAF, RAS, and PI3K pathways, together with the loss of function and suppression of PTEN, p53, and b-catenin in the less differentiated forms [5,6].

Recent progress in thyroid cancer biology revealed a certain degree of intratumoral heterogeneity [7,8,9,10], highlighting the coexistence of cellular subpopulations with distinct proliferative capacities and differentiation abilities, whose hierarchical organization is fundamental to the maintenance of the malignant phenotype [11]. Similarly to other solid tumors [12,13,14,15,16,17], a rare subpopulation of cells called cancer stem-like cells (CSCs) is hypothesized to drive the TC heterogeneity and contribute to the metastatic potential and therapy resistance [18,19,20,21]. CSCs exist predominantly in different specific tumor niches, where the dynamic equilibrium within cell-intrinsic and cell-extrinsic factors derived by the tumor microenvironment allow for the maintenance of the stem-like phenotype, which is characterized by a lack of tissue-specific differentiation, slow-cycling rate, quiescence, and theoretically unlimited self-renewal abilities [13,22,23].

In recent years, the discovery of thyroid CSCs uncovered the inadequacy of the “classical” carcinogenesis model and prompted further knowledge on the TC complex microenvironment.

## 2. Thyroid Cancer and CSCs

### 2.1. Thyroid Carcinogenesis and CSCs Origins

**Figure 1:**
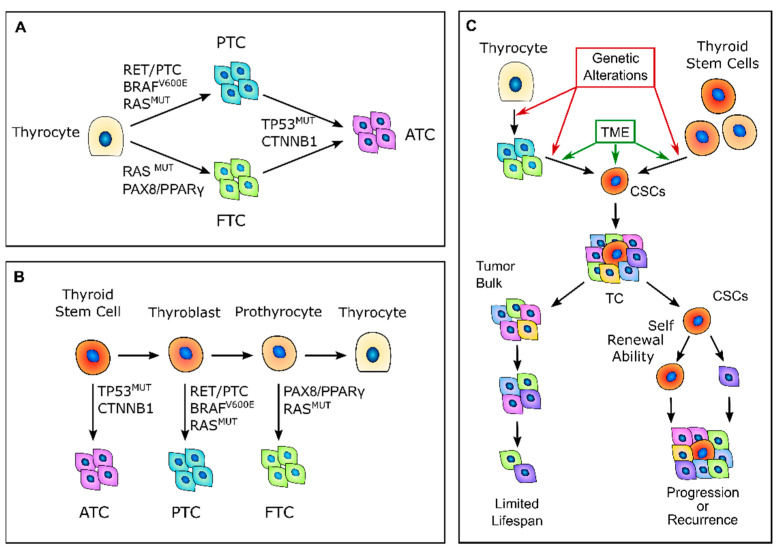
Thyroid carcinogenesis models. (**A**) Description of the classic multistep carcinogenesis model: the gradual accumulation of genetic alterations in normal thyrocytes leads to the transformation into cancer cells and to the acquisition of subsequently less differentiated and more aggressive phenotypes. Mutations in driver genes, such as *BRAF* and *RAS* or *RET/PTC* and *PAX8/PPARγ* rearrangements, give rise to the well-differentiated papillary thyroid cancers (PTCs) and follicular thyroid cancers (FTCs), while the acquisition of *TP53* and *CTNNB1* mutations leads to the transformation in anaplastic thyroid cancers (ATCs). (**B**) Fetal stem cells’ origin model: thyroid cancer cells are derived from normal stem cells or precursor cells of fetal origin that acquire transforming mutations. These genetic alterations confer proliferative advantages and prevent fetal thyroid cells from differentiating. Less differentiated stem cells give rise to ATCs, while the more differentiated thyroblasts and prothyrocytes give rise to PTCs and FTCs, respectively. (**C**) Cancer stem-like cells’ (CSCs) origin model: CSCs with high tumorigenic activity and increased ability to self-renew originate from either normal stem cells through a transformation process or from differentiated cancer cells as the result of a dedifferentiation process. The transition of stem cells into mature cancer cells is stimulated by the different tumor environment that is present outside the stem niches. Mature cells cannot sustain tumor progression, while CSCs can reconstitute and sustain tumor growth. TME, tumor microenvironment; TC, thyroid cancer.

According to the classic multistep carcinogenesis model (Figure 1A), TC cells arise from the gradual accumulation of genetic alterations within normal thyroid epithelial cells, leading to uncontrolled proliferation and an invasive phenotype [24,25]. Thus, PTC and FTC are the results of randomly occurring genetic alterations, such as *BRAF* and *RAS* point mutations or the more complex *RET/PTC* and *PAX8/PPARγ* rearrangements. The sequential accumulation of further genetic alterations, particularly the inactivating mutations of *TP53* and *CTNNB1*, can then give rise to ATC [26]. These events come with the dedifferentiation process that occurs as the cancer cells acquire the neoplastic phenotype, with a marked epithelial-to-mesenchymal transition (EMT), which is a process that finally results in CSCs’ phenotype acquisition [27,28]. Nevertheless, this model has some intrinsic limitations. While the mature thyroid follicular cells have a low proliferation rate, intrinsically limiting the accumulation of multiple mutations [29], the introduction of large-scale genome sequencing techniques revealed that PTC and FTC already have much more complex genetic alterations than what the classical multistep model can explain [30,31,32].

In 2005, Takano et al. [26] proposed that TC cells are derived from normal stem cells or precursor cells of fetal origin that survive in the mature gland rather than from differentiated thyroid follicular cells [26,33] (Figure 1B). According to this model, normal fetal thyroid stem cells, which express oncofetal fibronectin but none of the markers that are typical of differentiated thyroid cells, give rise to ATC. Thyroblasts, which express both oncofetal fibronectin and the differentiation marker thyroglobulin (Tg), give rise to PTC. Finally, prothyrocytes, which are the more differentiated cell type, should give rise to FTC and follicular adenoma [33]. In this model, genetic alterations confer proliferative advantages and prevent fetal thyroid cells from differentiating. However, there is no explanation regarding how quiescent thyroid stem cells acquire such genetic alterations or about the coexistence of cellular subpopulations with different degrees of differentiation. The evidence that a cancer cell population is heterogeneous and that molecular alterations are not present in the whole tumor bulk finally brought about the CSC hypothesis for TC. This hypothesis was first established by the previous observation that leukemia may contain hierarchical multi-lineage cells [34]. In this perspective, some authors hypothesized that TC may be a CSC-driven disease [26,35,36], with only a subset of cancer cells that possess high tumorigenic activity, with increased ability to self-renew and produce progenitor cells that can reconstitute and sustain tumor growth [1] (Figure 1C). The transition of stem cells into mature cells is stimulated by growth factors and cytokines present in the microenvironment outside the stem niche [25]. According to this view, CSCs may originate from either normal stem cells through a transformation process or from differentiated cancer cells as the result of a dedifferentiation process [35]. The clinical implication of the CSC model may give rise to important effects for both the diagnosis and treatment of TC, especially for the management of poorly differentiated, recurrent, or rapidly growing diseases that are refractory to radioactive iodine (RAI) therapy. In this view, the eradication of all CSCs may arrest tumor growth, whereas the failure to eliminate CSCs will eventually lead to tumor relapse [37].

### 2.2. Thyroid CSC Identification

Nowadays, CSC identification relies mostly on the identification of stemness biomarkers, together with specific in vitro and in vivo assays (Table 1).

In vitro assays aim to demonstrate the self-renewal abilities of the CSCs and comprise thyrosphere formation assays, limiting–diluting assay, serial colony formation, and differentiation assays. Because normal thyroid stem cells can be grown as sphere-like cellular aggregates in a specialized stem cell culture medium, the multicellular three-dimensional (3D) spheroids assay is the best-studied methodology to determine the clonality and multipotency of putative thyroid CSCs [1,38]. Indeed, the ability to generate spheres in serum-free medium, even after serial passages, indicates that the cells have an extensive capacity for self-renewal and should be able to recreate a heterogeneous tumor cell population and recapitulate the primary tumor morphology [39]. Moreover, different from two-dimensional monolayer cultures, tumor spheroids create intercellular contacts and usually display low values of nutrients, oxygen, and glucose, generating a hypoxic core in the center of the 3D structure, thereby imitating the natural environment of solid tumors [40]. Researchers have also established a colony-forming assay in which cells are cultured in a semisolid methylcellulose medium that recapitulates the extracellular matrix (ECM). This assay allows the clonal progeny of a single cell to grow as a distinct cluster or colony and monitors anchorage-independent growth, which is a key property of cancer cells.

The most definitive way to assess putative CSCs is to inject these cells into immunocompromised mice to verify their ability to develop tumors over time [41]. In particular, the serial transplantations of cells that were isolated from secondary and tertiary xenografts allow for defining their long-term tumorigenic potential, as well as their self-renewing ability [42]. A further enhancement of this approach involves combining these serial transplantations with limiting–dilution assays to determine the minimum number of cancer stem cells that are required for tumor formation and to confirm that tumor size is positively correlated with the number of cells injected [1]. Moreover, the ability of tumor initiation can be more accurately evaluated using an orthotopic transplantation to mimic the tumor environment as closely as possible [39].

**Table 1:** Markers that are used to identify thyroid CSCs.

Markers	Functions	References
aldehyde dehydrogenase (ALDH) activity (ALDEFLUOR)	Used to isolate CSCs based on their elevated ALDH activity via positive flow cytometry selection	[21,41,43,44,45,46,47]
CD133 (prominin-1)	CD133^+^ cells express stemness genes (*POU5F1*, *SOX2*, and *NANOG1*), drug-resistance genes (*ABCG2*, *MDR1*, and *MRP*), and a low expression of thyroid differentiation markers.	[47,48,49]
CD44^+^/CD24^−^ phenotype	CD44+/CD24− subpopulation of cells with tumorigenic potential identified by flow cytometry positive selection	[47,48,50]
Side population (SP) cells	Ability to exclude DNA-binding dye Hoechst 33342 via ABC family of transporters; they export anticancer drugs when overexpressed in tumor cells	[15,51,52]
Stem cell transcription factors (OCT-4, SOX2, NANOG)	Highly enriched markers in cell populations with stemness properties	[32,39,48,49,52,53,54]
EMT-promoting pathways (Notch-1, Wnt signaling, Sonic hedgehog protein)	Pathways involved in promoting self-renewal ability and tumorigenic potential	[39,53,54]

Many studies have been carried out to identify the specific biomarkers of thyroid CSCs in the three histopathological TC variants. 

Evaluating the enzymatic activity of aldehyde dehydrogenase (ALDH) is a well-known approach for identifying putative CSCs. Indeed, high levels of ALDH activity are present in stem and progenitor cells and seem to be related to their resistance to chemotherapy. Todaro et al. [21] were the first to isolate CSCs from primary thyroid tumors using ALDH activity. They demonstrated that the three histopathological TC variants expressed a small population of cells with tumorigenic potential, elevated ALDH activity, and unlimited replication ability [21]. This subpopulation of cells (1.2–3.5%) of the whole tumor was ALDH^high^ and was able to form thyroid spheres when expanded in vitro in serum-free conditions, as well as create sequential tumor xenografts in immunocompromised mice model [21]. Another putative CSCs marker is prominin-1, also called CD133, which is a five transmembrane domain glycoprotein with unknown function that behaves as a stemness marker in many normal and tumor cells. In TC, Tseng et al. [49] isolated CD133^+^ cells from ATC primary tumors and ATC cell lines. The CD133^+^ cells expressed stemness genes, such as POU class 5 homeobox 1 (*POU5F1*), sex-determining region Y-box 2 (*SOX2*), and *NANOG1*, as well as drug-resistance genes (*ABCG2, MDR1*, and *MRP*). These cells were also chemoresistant and formed thyrospheres in vitro and tumors in vivo [49].

Ahn et al. [50] identified CD44 and CD24 expression in a small percentage of cells with tumorigenic potential in PTC cell lines and human primary samples. They observed that this subset of cells with tumorigenic capability expressed high levels of CD44, but no expression was detected for CD24 (CD44^+^/CD24^−^) [50]. Moreover, these CD44^+^/CD24^−^ cells expressed the stem cell markers OCT4 and POU5F1 and had a low expression of differentiation markers [50].

To further identify specific thyroid CSCs markers, Shimamura et al. [47] performed a comprehensive analysis of multiple markers (CD13, CD15, CD24, CD44, CD90, CD117, CD133, CD166, CD326, and ALDH activity) on eight thyroid cancer cell lines and then evaluated their ability to form thyrospheres in vitro and tumors in vivo. Their results suggest that ALDH^pos^ and CD326^high^ subpopulation of cells showed higher sphere-forming ability and both self-renewal and differentiation capability, generating homogeneous and heterogeneous cell populations. However, even if ALDH activity and CD326 expression are reliable candidates for detecting thyroid CSCs, they are not universal [47].

Another method to detect CSCs is the side population (SP) assay. This identifies a small subpopulation of cancer cells that is able to exclude the DNA binding dye Hoechst 33342 through the adenosine triphosphate-binding cassette (ABC) family of membrane transporters, which is also responsible for the anticancer drug export and therapy resistance of CSCs [15].

SP cells have been identified in different TCs: they presented a primitive morphology, with a high nuclear-to-cytoplasmic ratio, the ability to undergo thyrosphere formation, and expressed typical stem cell markers, such as OCT-4, NANOG, and SOX2, but no markers of thyroid differentiation [51,52].

Mitsutake et al. [54] also found a very small portion of SP cells in human thyroid cancer cell lines. The detection of putative thyroid CSCs can also be supported by the evaluation of the expression of biomarkers belonging to self-renewing control pathways, such as Wnt/β-catenin, Sonic hedgehog protein, and Notch1, which are also responsible for the EMT process regulation [54]. Indeed, there is a strong correlation between EMT markers’ expression and the presence of CSCs in TC [28,55]. For example, a loss of E-cadherin is associated with the expression of CD44, CD133, and Nestin, while Snail1 and vimentin upregulation is associated with ALDH expression [56,57,58].

## 3. Tumor Microenvironment and CSC Maintenance

**Figure 2:**
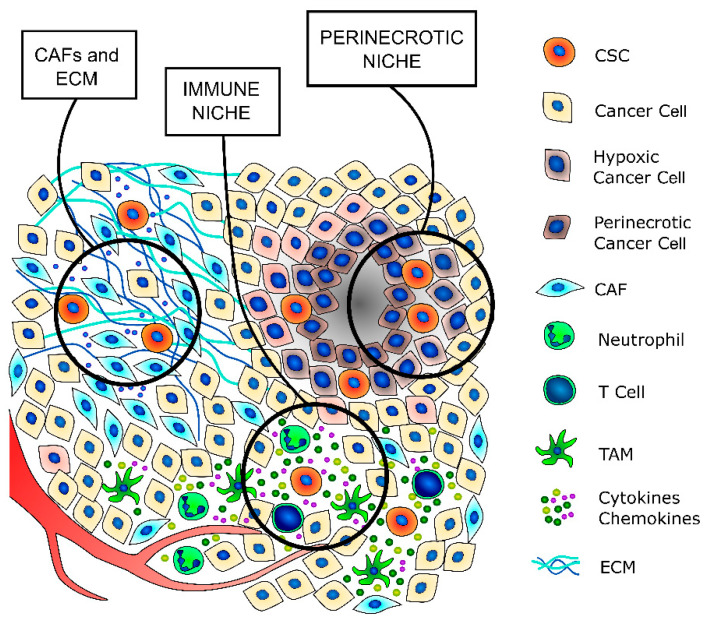
TC stem cell niches. CSCs principally exist in tumor areas with specific microenvironmental conditions, the so-called stem cell niches. The cancer-associated fibroblasts (CAFs) secrete a thicker extracellular matrix (ECM) and different prostemness soluble factors that induce the acquisition of a stem-like phenotype by cancer cells and promote and support the survival and self-renewal abilities of already existing CSCs. The immune niche is composed of all the different immune cells that contribute to regulate the CSCs’ phenotypes, such as T lymphocytes, tumor-associated macrophages (TAMs) and neutrophils. Indeed, immune cells secrete a wide variety of cytokines and chemokines that support the maintenance of the stem-like phenotype. The hypoxic niche contributes to CSCs’ phenotype maintenance, mainly through the induction of hypoxia-inducible factors (HIFs), whose activation deeply influences the paraphysiological adaptations to the changes of the tumor microenvironment. Indeed, increasing evidence indicates that HIFs are one of the main regulators of CSC subpopulation maintenance, not only by stimulating an increase in the number of CSCs but also by enhancing the stem-like phenotype of dedifferentiated cancer cells.

Cancer stem cells are a small subpopulation that principally exists in tumor areas with specific microenvironmental conditions, the so-called stem cell niches, which are constituted by different stromal cell types, including a vascular system, mesenchymal and immune cells, the ECM, and soluble factors [13,59] (Figure 2 and Table 2). The stromal cells and the substances that they secrete are fundamental to maintaining the CSCs in a quiescent state and regulating their self-renewal and differentiation through the modulation of several signaling pathways [59]. The principal regulators of thyroid CSCs are the cancer-associated fibroblasts (CAFs) and the matrix secreted from them, the local variations in nutrients and oxygen distribution, mainly due to tumor fibrosis and altered vasculature growth that may create specific hypoxic niches, and immune cells, such as tumor-associated macrophages (TAMs) and mast cells (MCs), which secrete important paracrine factors.

### 3.1. CAFs, Extracellular Matrix, and Desmoplasmic Reaction in TC

As with many other solid tumors of glandular origin, the more aggressive thyroid cancers are characterized by a pronounced desmoplastic stromal reaction [100,101,102,103,104,105,106,107,108], whose major components are CAFs and the ECM that they secrete (Figure 2) [107,109,110]. Indeed, different studies revealed that CAFs often secrete an ECM that is rich in collagen 1, which has been associated with tumor progression, metastatization, and therapy resistance [32,92,107,111,112,113,114,115,116].

In many cancer types, the crosstalk between the tumor and stromal components is fundamental for the establishment and maintenance of permissive niches that promote cancer progression and therapy resistance [117,118]. In fact, the interactions between cancer cells and CAFs often involve molecules such as CD44, thrombospondin-1, osteopontin, fibronectin, and integrins that are also involved in the induction and maintenance of CSCs’ phenotypes [50,95,96,109,110,119,120,121,122,123] (Table 2). Moreover, a specific subpopulation of CAFs that effectively secrete pro-stemness factors have recently been identified [61,82,87,88,124,125,126]; these pro-stemness factors can induce the acquisition of a stem-like phenotype by cancer cells in normal conditions and promote and support the survival and self-renewal abilities of already existing CSCs after different stresses, such as anticancer therapies [127,128,129,130]. 

Although in recent years, the relationships between CSCs, CAFs, and the ECM have been deeply explored, only a few studies have investigated this topic regarding thyroid cancer. 

In 2010, Nucera et al. demonstrated that the downstream pathways activated by BRAFV600E are crucial for ECM remodeling, where they identified thrombospondin 1 as one of the main effectors, together with CD44, fibronectin cathepsin B, and TGFb1 [131].

Further studies revealed that collagen 1 and lysyl-oxidase (LOX, a collagen fiber crosslinker) expressions are associated with less differentiated TC types and a poor overall survival rate; these alterations are the result of BRAF activation and/or PTEN loss, which promote the formation of a fibrotic tumor stroma that is rich in CAFs and collagen 1, facilitating tumor progression [117,132]. All these effects are mostly due to the fact that TC cells with specific genetic alterations secrete peculiar soluble factors that are able to activate the nearby fibroblast, inducing the changes in metabolism and phenotype that are typical of CAFs [131,133]. In turn, activated CAFs secrete soluble factors that modulate TC cells’ proliferative and invasion potentials [133].

Thus, it seems that the well-characterized paracrine loop between CAFs and cancer cells that is definitely important for the definition of specific niches in which CSCs are mainly localized also exists in TC; further studies are, however, needed in this direction. 

### 3.2. Hypoxic Niche

In many cancer types, hypoxia is a hallmark of malignancy and progression, as solid tumors often outgrow the vasculature, and the vasculature itself can be aberrant [134,135] (Figure 2). At the cellular level, hypoxia induces a complex coordinated response that deeply influences the paraphysiological adaptations to the changes in the tumor microenvironment, which are modifications that are often also responsible for therapy resistance [136,137,138].

The main players in the hypoxia response are two transcription factors, namely, hypoxia-inducible factors 1 and 2 (HIF-1 and HIF-2, respectively). HIF protein activity is finely regulated by the turnover of its oxygen-dependent alpha subunits (HIF-1α, HIF-2α, and HIF-3α) [135,139]. Upon exposure of the cells to hypoxia, the HIFα subunits are stabilized, translocate in the nucleus, and induce the transcription of different target genes that regulate cell metabolism, proliferation, invasive potential, and therapy resistance [140,141,142].

Indeed, increasing evidence indicates that HIFs are one of the main regulators of CSCs subpopulation maintenance, not only by stimulating an increase in the number of CSCs but also by enhancing the stem-like phenotype of dedifferentiated cancer cells [119,134,143,144,145]. HIF-1α or HIF-2α overexpression and their interplay in CSC maintenance have been observed in many cancer types, such as glioblastoma, colon, breast, lung, pancreatic, and ovarian cancers [22,146,147,148,149,150,151,152]. 

Although necrotic or hypoxic areas are uncommon in well-differentiated thyroid cancers, they are frequently found in the more aggressive anaplastic ones [3,153]. Moreover, it is known that HIF1α activity is also induced in non-hypoxic conditions by the hyperactivation of PI3K and RAS/RAF/ERK pathways, which is a direct consequence of genetic alterations that are common in thyroid cancer, such as PTEN deletion and RAS and BRAF mutations [154,155,156,157,158]. Overexpression of HIFs has been associated with an advanced tumor grade and distant metastasis in thyroid cancer [155,159].

Different studies demonstrated that the expression of HIF1 and its target genes, GLUT1 and VEGF, correlate with the TC grade and type [160,161,162,163,164]. While PTC and FTC showed only focal HIFs expression, PDTC and ATC presented a diffuse expression, which may be the result of a specific combination of tumor genotype and microenvironment alterations induced by diffusion-limited chronic hypoxia [155]. Up till now, no data exists on HIF2 expression in TC, but in vitro experiments demonstrated that HIF2 activation may be induced by the same stimuli that regulate HIF1, and its inhibition greatly affects thyroid cancer cell proliferation in hypoxic conditions [159,165].

### 3.3. Immune Niche

CSCs have a dual relationship with the immune components of the microenvironment. On one side, they are able to escape the attacks of immune cells against bulk cancer thanks to their quiescent state, low immunogenicity, and ability to recruit immunosuppressive cells [166,167]. On the other hand, immune cells secrete a wide variety of cytokines and chemokines that support the maintenance of the stem-like phenotype [168,169] (Figure 1 and Table 2). All the different immune cells that contribute to regulating the CSCs phenotype maintenance, such as T regulatory lymphocytes (T-regs), TAMs, and MCs, compose the so-called immune niche.

The immune evasion mechanism of TC cells is mainly based on the downregulation of MHC class I molecules, the upregulation of B7 homolog I (B7-HI), and the upregulation of HIF-1α. Angell et al. [170] found that the expression of human histocompatibility antigen (HLA)-ABC and β2-microglobulin in PTC was significantly reduced compared with normal thyroid tissue, and the proportion of tumor-infiltrating lymphocytes (TILs) was also decreased. In PTC, the B7-H1 protein and mRNA levels are strongly associated with tumor aggressiveness: the higher the B7-H1 expression level, the stronger the tumor aggressiveness [171]. Moreover, the hypoxic-like environment characterized by HIF-1 activation, which promotes CSC maintenance, also possesses an immune-suppressive effect that enhances tumor escape [172].

T-regs are highly enriched in the tumor microenvironment (TME), where they reduce the antitumor immune response. Some studies have suggested that T-regs can efficiently migrate into tumors in response to chemokines (e.g., chemokine receptor 4 (CCR4)-CC motif ligand 17/22 (CCL17/22), CCR10-CCL28, and CXC chemokine receptor 4 (CXCR4)-CXC motif ligand 12 (CXCL12)) that are expressed on the stroma and tumor cells and can be associated with a poor prognosis in patients [173,174].

Cytotoxic T lymphocytes (CTLs) are another subpopulation of T lymphocytes that possess the ability to kill target cells. Regarding thyroid cancer, French and colleagues [175] found that a low concentration of CD8^+^ T cells and a reduced ratio of CD8/Foxp3^+^ T cells was correlated with a larger tumor diameter in PTC patients. In fact, a reduced number of CD8^+^ T cells diminishes the lethality to cancer cells and accelerates their rapid growth and invasiveness. Natural killer (NK) cells destroy pathogenic cells, mainly by secreting perforin and granzyme, expressing Fas ligand (FasL), and destroying their targets through antibody-dependent cell-mediated cytotoxicity (ADCC) [176]. Studies have reported that CSCs secrete immunosuppressive factors, such as transforming growth factor-β (TGF-β), indoleamine2,3-dioxygenase (IDO), arginase-1, and interleukin 6 (IL-6), which reduces the expression of NK cell surface-activated receptors and result in a decreased number and quality of NK cells [86,93,97,177].

MCs secrete cytokines, such as tumor necrosis factor α (TNF-α) and IL-8 to stimulate immune tolerance and enhance tumor progression [78,79,93,99]. The role of MCs has been widely studied in TC and its density has been positively correlated with cancer aggressiveness [130]. Indeed, Visciano et al. [79] found that TC cells activate MCs to produce chemokines, such as Il-6 and TNF-α, which in turn induce the EMT and a stem-like phenotype in TC cells.

TAMs are the most abundant population of tumor-infiltrating immune cells in the TME and have strong plasticity, as they can switch between proinflammatory (M1) and anti-inflammatory (M2) phenotypes [178,179].

The frequency of TAM infiltration varies between TC subtypes, increasing with dedifferentiation and culminating in ATCs, where TAMs represent up to 50% of all immune cells [180]. In all TCs, TAM infiltration has been invariably correlated with poor prognosis, large tumor size, capsular invasion, extra-thyroid tumor extension, lymph node metastasis, and decreased survival [181,182,183]. 

Indeed, TAMs secrete a wide variety of cytokines and chemokines, such as CXCL-8, TGF-β, and CCL-2, which influence CSC survival [61,71,79,86,184,185]. 

### 3.4. Exosomes in CSC Niches

Exosomes are extracellular vesicles with an endosomal origin that are produced by the different cell types present in the CSC niches [186,187]. Indeed, the intercellular communication mediated by exosomes plays a fundamental role in tumor development and aggressiveness [188].

In thyroid cancer, the CSCs produce exosomes containing different long non-coding RNAs (lncRNAs) that promote the EMT and the acquisition of a stem-like phenotype of the bulk cancer cells and induce a pro-metastatic phenotype [28,189,190,191]. Moreover, patients with metastatic PTC have significantly higher levels of circulating exosomal miRNAs and hypoxic PTC cells can secrete exosomes that modulate the expression of TGF-β and collagen isoforms, enhancing the tumoral angiogenesis [192]. The exosomes secreted by CSCs also modulate the polarization of TAMs toward the M2 phenotype and suppress NK cell activity, promoting an immunosuppressive environment in CSC niches [193,194,195,196]. In turn, CAFs and TAMs also release exosomes that contribute to the regulation of the TME [187,197,198]. 

## 4. Genetic Alterations, TME, and CSCs

Many of the different genetic alterations present in TC cells not only confer a proliferative advantage to the cell themselves but also deeply influence the surrounding microenvironment and the survival of CSCs.

The most studied alteration in this regard is BRAFV600E. Mutated cells have an altered expression of factors that are crucial for ECM remodeling, such as thrombospondin 1, CD44, fibronectin, cathepsin B, TGF-β1, collagen 1, and LOX [117,131,132]. Indeed, the ECM of BRAFV600E TCs has a composition and stiffness that promote the EMT of TC cells and enhances the stem-like phenotype of CSCs. Moreover, the alterations induced by BRAF hyperactivation also induce a more acidic TME that also contributes to the induction of an undifferentiated cell phenotype [199].

Moreover, BRAFV600E also significantly induces HIF1a in a hypoxia-unrelated way, and through TIMP-1 activation, synergizes with HIF1A itself to promote metastatic potential and a stem-like phenotype [200,201]. Similar alterations are also found in TC with PTEN loss, though fewer studies support these findings [117]. 

Alterations in p53 activity that are usually found in less differentiated TCs can deeply influence the CSCs phenotype and maintenance in different ways, from metabolic reprogramming to immune evasion. Even if p53 loss-of-function mutations are characteristic only of the less differentiated TCs, the proinflammatory TME induced by bulk cancer cells can suppress p53 function with various mechanisms. For instance, the activation of pathways such as NOTCH, WNT/b-catenin, and Hedgehog contribute to CSC stemness maintenance by suppressing p53 expression [202,203,204,205,206].

Indeed, p53 activity is critical for the maintenance of cell proliferation and differentiation, where the loss of p53 functionality promotes the dedifferentiation and maintenance of CSCs [207]. In addition, p53 suppression has also been reported in the thyrosphere generated from wild-type p53 TCs [208], and the inhibition of p53 is also fundamental in the reprogramming process that allows for the generation of induced pluripotent stem-cells (iPS) cells [209,210,211,212]. 

Another mechanism by which p53 loss promotes stemness is the upregulation of Twist1 and Snail2 expression, which are two important regulators of the EMT process that promote the generation of CSCs through the dedifferentiation of cancer cells [213,214]. Moreover, p53 can also regulate CD44 expression by modulating its alternative splicings through the RNA-binding protein ZMAT3 [215].

Furthermore, a loss of p53 functions promotes the metabolic switch from cellular respiration to glycolysis known as the Warburg effect [216,217]. This is fundamental for the survival of CSCs in the altered tumor microenvironment, and especially in the hypoxic niches.

Lastly, epigenetic alterations also play a role in CSC maintenance, but this area has been scarcely investigated in TC. A significant number of genes with abnormally methylated promoters in TC are involved in the regulation of the MAPK pathways controlled by RAS, BRAF, and PI3K, and can act as regulators of the EMT [218,219,220]. In addition, in FTC, hypermethylation of the E-cadherin promoter has been reported and was hypothesized to be a further mechanism of the EMT [221,222]. A similar mechanism is also responsible for the suppression of thyroid differentiation markers, such as NIS and TTF1 [223,224].

## 5. Therapeutic Targeting of CSCs and TME Crosstalk

**Figure 3:**
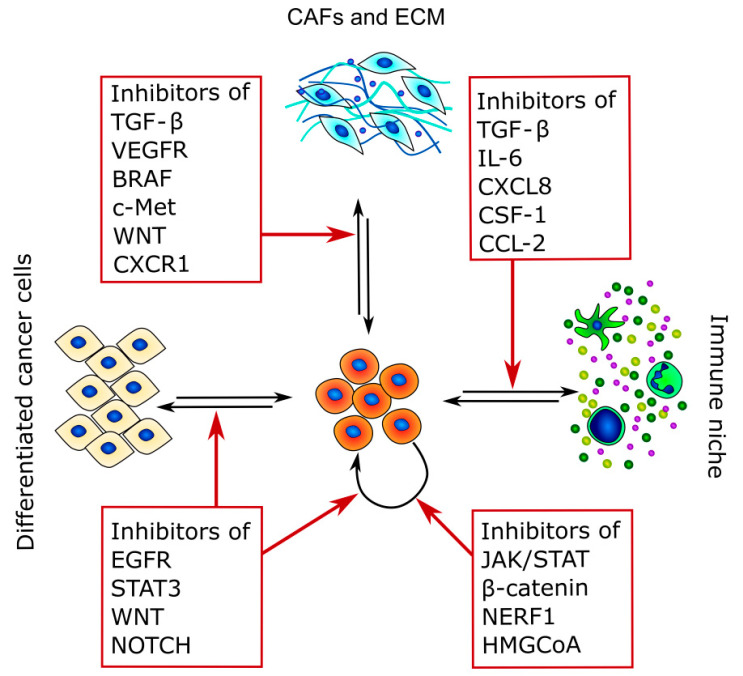
Therapeutic targets of CSCs and TME crosstalk. The figure summarizes the main pathways that can be targeted and highlights the main interactors of CSC crosstalk in which they are involved.

Because CSCs are the main cause of therapy resistance and disease relapse, in recent years, different strategies to target these cancer subpopulations have been developed. Indeed, CSC-targeting therapies rely mostly on three different strategies: the inhibition of CSC stem factors, the modulation of CSCs and TME crosstalk, and the promotion of CSC differentiation [225] (Figure 3 and Table 3). 

The first approach is the use of different molecules that directly target the pathways necessary for CSC survival, alone or in combination with more classical anticancer drugs [226,227].

In this sense, tyrosine kinase inhibitors (TKIs) are the more studied compounds, as they can inhibit different pathways that are involved in either cell proliferation or stemness acquisition. TKIs against the EGFR pathway are the most studied, as many CSC characteristics, such as quiescence, glycolytic metabolism, and immunosuppressive activity, are modulated by EGFR and its downstream target STAT3 [228,229]. Indeed, STAT3 is required for the survival of CD133^+^ TC cells, and inhibition of this transcription factor suppresses CSC tumorigenesis in vitro and in vivo [49].

Another pathway that has recently been explored for CSC targeted therapy is the Wnt/β-catenin one, together with its interactors NHERF1 and PTEN [230]. 

For example, Defactinib, which was developed first as a FADK1/2 inhibitor, was shown to directly target CSC survival through the modulation of β-catenin localization and activity [231]. Moreover, different inhibitors of Wnt and β-catenin are actually undergoing phase I and II clinical trials.

Besides TKIs, different compounds that were previously developed for other diseases had shown in vitro and in vivo efficacy against CSCs, principally by modulating their metabolism. For example, Lovastatin, which is an inhibitor of hydroxymethylglutaryl coenzyme A reductase, is able to target CSCs in mammary tumors due to its intrinsic mechanism of action [232,233].

Similarly, other statins and metabolically active drugs, such as metformin and menadione, efficiently target CSC survival in different cancer types [234,235,236].

As CSCs are a highly heterogeneous population that exist in a dynamic equilibrium between different differentiation states, they could easily escape one of the targeted therapies mentioned above, thus explaining the therapeutic failure of some TKIs. Moreover, conventional anticancer therapies are not only not effective against CSCs but can also induce CAFs to secrete different chemokines that indeed support CSC survival, thus finally resulting in relapses that are more aggressive than the original tumor [129]. Nevertheless, CAFs are a more stable population that is easily identifiable, and at the same time, the paracrine factors that they secrete are a fundamental support for CSCs. For these reasons, the disruption of the crosstalk between CSCs and CAFs by directly targeting the molecular signaling pathways involved may be successful.

For example, a gastric CSC population was significantly suppressed by TGF-β inhibition [237,238]. Unfortunately, although TGF-β secretion is dysregulated by the frequent TC BRAFV600E mutation, no clinical studies have been performed for TC in this sense.

Moreover, as previous studies revealed that c-Met silencing inhibits the metastatic potential of TC CSCs [21], this could be another potential target. Indeed, c-MET and β-catenin pathways are both regulated by CAF-secreted HGF and WNT [239,240], and their inhibitors are under investigation in phase I clinical trials [241,242].

The inhibition of IL-6 activity by specific antibodies in combination with chemotherapy successfully induced an almost complete regression in a PDX model of breast cancer by interrupting the inflammatory loop between the IL-6 and STAT-3 responsible for the EMT and stemness maintenance [243]. Besides IL-6, molecules that act against its downstream pathway are another opportunity for directly targeting CSCs and simultaneous inhibition of CSC–CAF crosstalk. Moreover, different molecules that disrupt the CSC–immune cells crosstalk are actually under investigation. For example, compounds targeting CXCR4 effectively induced remission in hematopoietic cancers.

## 6. Conclusions

Although TC is a manageable disease in the majority of the cases, the more aggressive and less differentiated types are still highly lethal diseases. The discovery of CSCs and the complex dynamics that exist in the tumor microenvironment and highly specialized niches may explain how TC subpopulations can survive different anticancer drugs, leading to disease recurrence and therapeutic failure. Despite the CSC–TME interplay being well studied for other cancer types, this field is still evolving for TC, with some important studies that identified the TC CSCs but with scarce knowledge of the TME complexity. The understanding of the heterogeneous biology of TCs will prompt the development of more specific therapies, which can be directed not only against the cancer cell bulk but also aimed at disrupting the crosstalk between CSCs and the different components of the TME, and finally allowing for the complete eradication of the disease.

## Figures and Tables

**Table 2:** Secreted factors that promote CSCs’ phenotypes and survival.

Factors	Cell Type	Action/Function	Pathways Involved	Other Cancers	References
CCL-2	CAFs, TAMs	Stimulates CSCs survival	Notch, NF-κB	Breast cancer	[60,61]
CCL-15	TCs	Recruit TAMs Promotes therapy resistance	CCR1		[62]
CXCL-1	CAFs	Promotes cancer cell stemness	IL-1a/JAK/STAT	Pancreatic ductal adenocarcinoma	[63,64,65]
CXCL-12	CAFs	Induces CSC markers expression	Wnt, PI3K/Akt	Colorectal cancer	[66,67,68,69,70]
CXCL-2	CAFs	Promotes cancer cell stemness	IL-1a/JAK/STAT	Pancreatic ductal adenocarcinoma	[65,71]
CXCL-8	TAMs, TCs	Promotes and maintains CSCs phenotype and induces EMT.	NF-κB, EGFR/RAS	Melanoma, ovarian cancer Colorectal cancer, non-small cell lung cancer	[71,72,73,74,75,76,77,78,79]
HGF	CAFs	Induces CSC markers expression	PI3K, Met	Colorectal cancer	[70,80]
IGF-2	CAFs	Promotes acquisition of stem-like phenotype	PI3K, TGF-β, Wnt, SHH	Non-small cell lung cancer	[48,81,82]
IL-6	CAFs, MCs	Promotes and maintains CSCs phenotype and induces EMT.	JAK/STAT, NF-κB	Breast cancer, pancreatic ductal adenocarcinoma	[65,79,83,84,85,86,87,88]
IL-8	CAFs, MCs	Promotes and maintains CSCs phenotype	FAK/AKT, Akt/Slug	Breast cancer, pancreatic ductal adenocarcinoma	[89,90,91,92,93]
OPN	CAFs, TCs	Supports the clonogenic capacity of CSCs Induces CSCs markers expression	Wnt, PI3K	Colorectal cancer	[70,94,95,96]
TGF-β	CAFs, TAMs, TCs	Induces CSCs markers expression	Wnt, PI3K	Colorectal cancer	[70,97]
TNF-α	MCs	Stimulate immune tolerance	CCL20 CXCL8/EGFR	Breast cancer, colorectal cancer	[93,98,99]

MCs: mast cells.

**Table 3:** Compounds that act against CSC-related pathways.

Pathways	Compound	Cancer Type	Clinical Trials
CD44	H90, P245, H4C4, RO5429083, SPL-108, AMC303	Ovarian, solid tumors	NCT01358903, NCT03078400, NCT03009214
CD133	BsAb	Glioblastoma	NCT03085992
Nanog	BBI503	Hepatocellular, advanced solid tumors	NCT02483247, NCT01781455, NCT02279719
EpCAM	Catumaxomabr (emovab)	Ovarian	NCT00189345
Notch	MK-0752, RO4929097, LY3039478, AL101, CB-103, BMS-906024	Pancreatic, breast, melanoma, hematologic malignancies	NCT00106145, NCT01098344, NCT00645333, NCT01196416, NCT01232829, NCT02836600, NCT02518113, NCT03422679 NCT01363817
Wnt	Vantictumab, Ipafricept	Pancreatic, ovarian, hepatocellular, breast	NCT02050178, NCT02092363, NCT02069145, NCT01973309, NCT02005315
Β-catenin	PRI-724, CWP232291	Pancreatic, hematologic malignancies	NCT01606579, NCT01764477, NCT01398462, NCT02426723
FAK	Defactinib/VS-6063, VS-4718	Lung, ovarian, non-hematologic	NCT01951690, NCT01778803, NCT02004028, NCT01849744
JAK/STAT	Ruxolitinib, AZD4205, SAR302503, BBI608	Breast, glioblastoma, hematopoietic malignancies, pancreatic	NCT01594216, NCT00952289, NCT03450330, NCT01523171, NCT02178956, NCT02315534, NCT02352558, NCT02231723
EGFR	Bevacizumab, Matuzumab	Breast, gastric	NCT01190345, NCT00215644
HIFs	PX478, Topotecan	Solid tumors, lymphoma, ovarian	NCT00522652, NCT01600573, NCT00194935, NCT02963090
AMPK	Metformin	Ovarian, lung	NCT01579812, NCT01717482
CXCR4	Plerixafor, BL-8040, BKT140, BMS-936564, LY2510924, USL311, AMD3100	Pancreatic, glioblastoma, hematologic malignancies	NCT02179970, NCT02907099, NCT02765165, NCT00512252
TGF-β	Galunisertib, LY3200882, Trabedersen, Fresolimumab, Vactosertib, NIS793	Prostate, colorectal, pancreatic, breast, melanoma, hepatocellular, glioblastoma	NCT02452008, NCT04031872, NCT00844064, NCT01959490, NCT00431561, NCT00356460, NCT02947165

## Data Availability

No new data were created or analyzed in this study. Data sharing is not applicable to this article.
